# LPA Promotes T Cell Recruitment through Synthesis of CXCL13

**DOI:** 10.1155/2015/248492

**Published:** 2015-08-03

**Authors:** Weili Hui, Chenqi Zhao, Sylvain G. Bourgoin

**Affiliations:** Rheumatology and Immunology Research Center, CHU de Québec Research Center and Faculty of Medicine, Laval University, 2705 Laurier Boulevard, Québec, QC, Canada G1V 4G2

## Abstract

Lysophosphatidic acid (LPA) is a bioactive phospholipid playing an important role in various inflammatory diseases by inducing expression and secretion of many inflammatory cytokines/chemokines. Here we report in a murine air pouch model of inflammation that LPA induced CXCL13 secretion in a time-dependent manner and with exacerbation of the response when LPA was administered after a pretreatment with TNF-*α*, a key inflammatory cytokine. LPA mediates recruitment of leukocytes, including that of CD3^+^ cells into unprimed and TNF-*α*-primed air pouches. CXCL13 neutralization using a blocking antibody injected into air pouches prior to administration of LPA into TNF-*α*-primed air pouches decreased CD3^+^ cell influx. Our data highlight that LPA-mediated CXCL13 secretion plays a role in T cell recruitment and participates in regulation of the inflammatory response.

## 1. Introduction

Lysophosphatidic acid (LPA) is a bioactive phospholipid with a simple structure containing a three-carbon glycerol backbone and a single acyl side chain that can vary in length and saturation [1]. By binding to and activation of its specific G protein-coupled receptors (GPCRs), LPA has been shown to evoke a great diversity of cellular responses, pointing out its important role in various physiological and pathophysiological situations [2, 3]. Increasing numbers of studies show that LPA plays a role in various inflammatory diseases [4–6]. Elevated LPA levels were detected in several biological fluids collected from different animal models of inflammation or patients with inflammatory diseases [2, 7, 8]. Increased expression of the LPA producing enzyme autotaxin (ATX) has also been reported in synovial tissues from patients with rheumatoid arthritis (RA) [8, 9]. Elevated expression of ATX and/or aberrant expression of LPA receptors were also found in several human malignancies [10]. LPA not only acts as a mediator implicated in cellular migration, growth, and immune cell chemokinesis, but also promotes directed cell motility indirectly by inducing cytokine/chemokine secretion [11]. CXCL8 is one of the leukocyte chemoattractant chemokines reported to be induced by LPA in fibroblast-like synoviocytes from RA patients [9], as well as various other cell types [12–15].

Chemokines play a key role in cellular trafficking of leukocytes during inflammation and immune surveillance [16]. CXCL13 is a CXC chemokine characterized as the sole B cell chemoattractant signal, originally named BLC and BCA-1 [17]. The only known receptor for CXCL13 is CXCR5, which exclusively binds CXCL13 [18]. CXCR5 is expressed by mature B cells [19], a subset of CD4+ and CD8+ T cells in secondary lymphoid tissue follicles [20], immature dendritic cells (DCs) [21], and macrophages [22]. The CXCL13-CXCR5 axis regulates T cell migration to the germinal centers in lymphoid tissues for early T-B cell collaboration and B cell activation [23], induction of migration of immature DCs [21], and maintenance of epithelial cell angiostatic activity [24]. Human CXCL13 is also reported to be an agonist of human CXCR3 receptor, which plays an important role in recruitment of activated T cells into secondary lymphoid tissues [25]. CXCL13 has been considered a putative diagnostic marker for some acute or chronic infectious and inflammatory diseases [26–30]. Increased CXCL13 production by osteoblasts from osteoarthritis patients in response to stimulation with IL-1*β* has been reported [31].

In the mouse air pouch model of inflammation activation of LPA1 and LPA3 receptors regulates leukocyte recruitment mainly through CXCL1 chemokine synthesis and its cognate receptor CXCR2 [11]. In this model TNF-*α*, which is a key inflammatory cytokine in autoimmune diseases such as RA, increases the expression of LPA1 and LPA3 in the air pouch lining tissue. By mimicking a proinflammatory environment, priming of air pouches or of human synovial fibroblasts with TNF-*α* exacerbates cytokine/chemokine secretion in response to LPA [9, 11]. Even though LPA induces the secretion of numerous cytokines/chemokines, whether LPA is able to recruit leukocytes including T and B cells to an inflammatory site through synthesis of CXCL13 has not been investigated. We used the murine air pouch model to assess the interaction between LPA, CXCL13, and lymphocyte recruitment after local pretreatment with TNF-*α*, which mimics severe inflammation* in vivo*. Neutralization of CXCL13 with a blocking antibody was also performed to determine whether LPA-mediated CXCL13 secretion regulates recruitment of leukocyte subsets into the air pouches. We demonstrate that LPA induces the recruitment of leukocytes including T lymphocytes into air pouches through a mechanism that is mostly dependent on CXCL13 synthesis.

## 2. Materials and Methods

### 2.1. Materials

Oleoyl-sn-glycero-3-phosphate (LPA) was purchased from Sigma-Aldrich Canada (Oakville, ON, Canada). Murine TNF-*α* was from PeproTech Inc. (Rocky Hill, NJ, USA). CXCL13 ELISA dual kit, rat anti-mouse CXCL13 antibody (rat IgG2A, clone 143614), control rat IgG2A (clone 54447), and Proteome ProfilerTM Mouse Cytokine Array Panel A were purchased from R&D Systems Inc. (Minneapolis, MN, USA). Anti-CD16/CD32, anti-mouse CD11b-eV450, and their matched isotype controls were from eBioscience (San Diego, CA, USA). Anti-mouse CD3e-APC, anti-mouse CD19-PE, and their matched isotype controls were from BD Bioscience (San Diego, CA, USA). All other reagents were obtained from Sigma-Aldrich Canada (Oakville, ON, Canada).

### 2.2. The Air Pouch Model

Female Balb/c (wild type) mice 6–8 weeks old (Charles River, St.-Colomban, Canada) were used to create air pouches. All experimental procedures carried out on mice were approved by the Animal Care Committee at Laval University and conformed to the Canadian Council on Animal Care standards and guidelines.

Air pouches were raised on the dorsum of mice by subcutaneous injection of 3 mL sterile air on days 0 and 3 as previously described [11]. Before the injection of air, mice were briefly anesthetized with isoflurane. On day 7, LPA (3 *μ*g) in 1 mL of phosphate-buffered saline (PBS) supplemented with 0.1% endotoxin-free delipidated bovine serum albumin (BSA) was injected into air pouches. TNF-*α* (50 ng) was injected into air pouches 16 h prior to stimulation with LPA or administration of the CXCL13 neutralizing antibody. To assess the impact of CXCL13 neutralization on LPA-induced leukocyte recruitment, the rat anti-mouse CXCL13 blocking antibody (10 *μ*g) was injected into the air pouch 15 min prior to stimulation with LPA. At specific times, mice were anesthetized with isoflurane and killed by asphyxiation using CO_2_. Air pouches were washed twice with 1 mL of PBS containing 5 mM EDTA and harvested pouch fluids were centrifuged 5 min at 3000 rpm. The supernatants were collected and kept at −80°C for later cytokine/chemokine measurements. Cell pellets were suspended in PBS-EDTA and counted using the Moxi mini automated cell counter (ORFLO, Hailey, ID, USA) prior to cell staining for flow cytometry analyses.

### 2.3. Flow Cytometry Analysis

For flow cytometry analysis, the cell pellets from each group of mice were suspended in flow cytometry staining buffer (eBioscience, San Diego, CA, USA) and the cells were pooled into one tube. The samples were then incubated with the anti-CD16/CD32 antibody (0.5 *μ*g/10^6^ cells) for 15 min on ice for Fc*γ*R blocking prior to cell staining with 0.1 *μ*g of anti-mouse CD11b-eV450, 0.1 *μ*g of CD3e-APC, and 0.1 *μ*g of anti-mouse CD19-PE for 45 min. Cell suspensions were then processed for FACS analysis using a FACSCalibur (Becton Dickinson, Mississauga, ON, Canada).

To prepare single cell suspension of splenocytes, the spleens from mice with inflamed air pouches were collected, mechanically disrupted, and passed through a strainer according to the manufacturer's instructions (BD Bioscience). FACS analysis used mouse splenocytes as positive controls for titration of anti-CD3 and anti-CD19 antibodies and gating of CD3^+^ and CD19^+^ cells.

### 2.4. Assessment of CXCL13 Secretion in the Air Pouch Lavage Fluids

The air pouch exudates from each treatment group (5–10 mice) were pooled and incubated with the Proteome Profiler Mouse Antibody Array Panel A according to the manufacturer's instructions for qualitative and semiquantitative analysis of cytokine/chemokine production by densitometry. Each pair of duplicate spots on the film represents a specific cytokine/chemokine. For accurate quantification of the levels of CXCL13 in air pouch lavage fluids from each mouse, a CXCL13 ELISA was performed according to the manufacturer's instructions (R&D Systems). Each sample was tested in duplicate and the results were compared with a standard curve that was generated using known concentrations of CXCL13. The dynamic range of the CXCL13 ELISA is 15.6 pg/mL–1000 pg/mL.

### 2.5. Statistical Analysis

Experiments were performed with 5–10 mice/group and results are expressed as mean ± SE of representative studies. All statistical analysis was performed using Prism 5.0 software. Statistical significance of the difference between samples of two different treatments was determined by *t*-test (two-tailed *P* value). For the time course studies, statistical significance between nontreated (NT) samples or samples treated at 0 h and those treated for the indicated time points was determined by one-way ANOVA, Dunnett's multiple comparison test. Multiple comparisons in the same experiment were made using one-way ANOVA, Bonferroni multiple comparison test. *P* values less than 0.05 were considered statistically significant.

## 3. Results

### 3.1. LPA-Mediated Release of CXCL13

**Figure 1:**
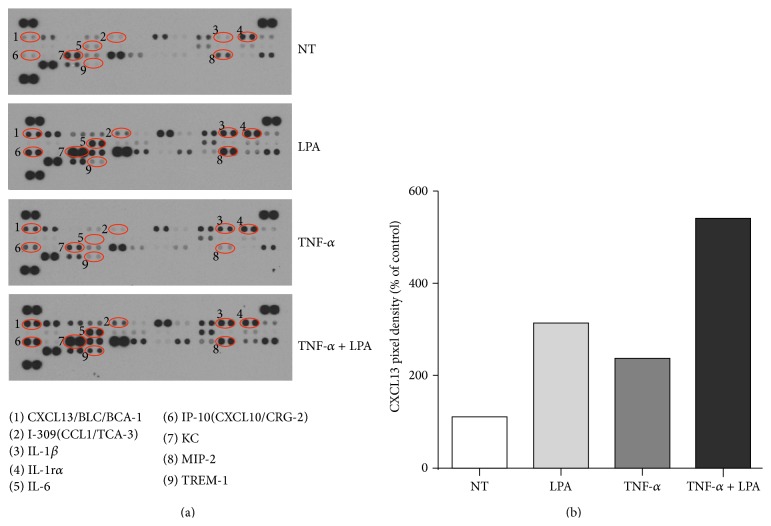
Effect of LPA on CXCL13 secretion in the murine air pouch with or without TNF-*α* pretreatment. (a) Six-day-old air pouches were produced in the dorsal skin of mice and injected with TNF-*α* or the vehicle for 16 h prior to stimulation with LPA for 2 h. The nontreated (NT) group was injected with vehicle only (PBS-BSA). The air pouch exudates (*n* = 3) were collected and pooled for qualitative analysis of cytokine/chemokine secretion using the Proteome Profiler Mouse Antibody Array Panel (a). (b) Normalized data representing CXCL13 pixel density are the mean from two independent experiments.

LPA injected into air pouches has been reported to induce the synthesis of multiple cytokines/chemokines including IL-6, IL-1*β*, IL-16, KC, IP-10, and MIP-2 [4, 11]. Whereas the chemokine CXCL1 (also named KC or Gro-*α*) plays a role in LPA-mediated leukocyte recruitment into the mouse air pouch, blocking CXCL1 or its receptor CXCR2 does not completely reduce leukocyte influx suggesting the involvement of other chemokines or inflammatory mediators [4, 11]. In this series of experiments we focused on LPA-induced CXCL13 secretion into the air pouches. As previously reported [11], injection of 3 *μ*g LPA into the air pouch for 2 hours increases the secretion of CXCL13 as assessed using a qualitative mouse Cytokine/Chemokine Antibody Array assay (Figure 1(a)). Pretreatment of the air pouch tissues with TNF-*α* (50 ng) for 16 hours also increased the levels of CXCL-13 in the air pouch exudates relative to mice injected with vehicle alone. The combined effect of TNF-*α* pretreatment prior to LPA stimulation enhances CXCL13 synthesis as estimated by densitometry (Figure 1(b)).

**Figure 2:**
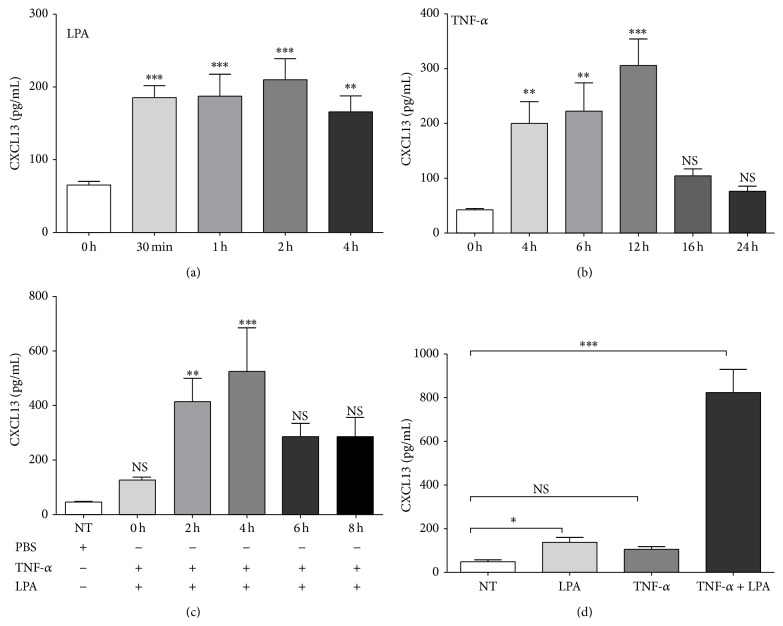
Effect of LPA and TNF-*α* on CXCL13 secretion in the air pouch. (a), (b) Kinetics of LPA and of TNF-*α*-mediated CXCL13 secretion. (a) LPA (3 *μ*g) or (b) TNF-*α* (50 ng) was injected into air pouches and air pouch exudates were collected at indicated times. (c) Kinetics of LPA-induced CXCL13 secretion in air pouches pretreated with TNF-*α*. TNF-*α* was injected 16 h before LPA stimulation. Air pouch exudates were collected at indicated times. (d) Comparison of LPA-mediated CXCL13 secretion in untreated and TNF-*α*-primed air pouches. TNF-*α* or vehicle was injected 16 h prior to administration of LPA for 2 h. Exudates were collected and cytokine/chemokine secretion was measured by ELISA. The nontreated (NT) groups were injected with vehicle only (PBS-BSA). Data are the mean ± SE from three independent experiments performed with at least five mice per group. ^*∗*^ *P* < 0.05, ^*∗∗*^ *P* < 0.01, and ^*∗∗∗*^ *P* < 0.001.

ELISA was then used to accurately quantify the kinetics of CXCL13 secretion (Figure 2(a)). The release of CXCL13 was significantly increased at 30 min after LPA stimulation and remained elevated up to 4 hours, the last time tested. TNF-*α* injected into the air pouches also induced CXCL13 secretion in a time-dependent manner (Figure 2(b)). A significant increase in CXCL13 secretion was observed at 4 hours and reached a maximum at 12 hours after TNF-*α* treatment, after which it declined. Although not statistically significant, a trend for higher levels of CXCL13 in air pouch lavage fluids at 16 hours following TNF-*α* treatment was observed compared to mice injected with vehicle alone (Figures 2(b) and 2(c)). When air pouches were pretreated with TNF-*α* for 16 hours, LPA induced robust secretion of CXCL13, which peaked at 2–4 hours after LPA stimulation (Figure 2(c)). TNF-*α* injected into the air pouches prior to LPA stimulation for 2 hours greatly potentiated CXCL13 secretion compared to mice injected with TNF-*α* alone or LPA alone (Figure 2(d)).

### 3.2. LPA Recruits Various Leukocyte Subtypes into the Air Pouch

**Figure 3:**
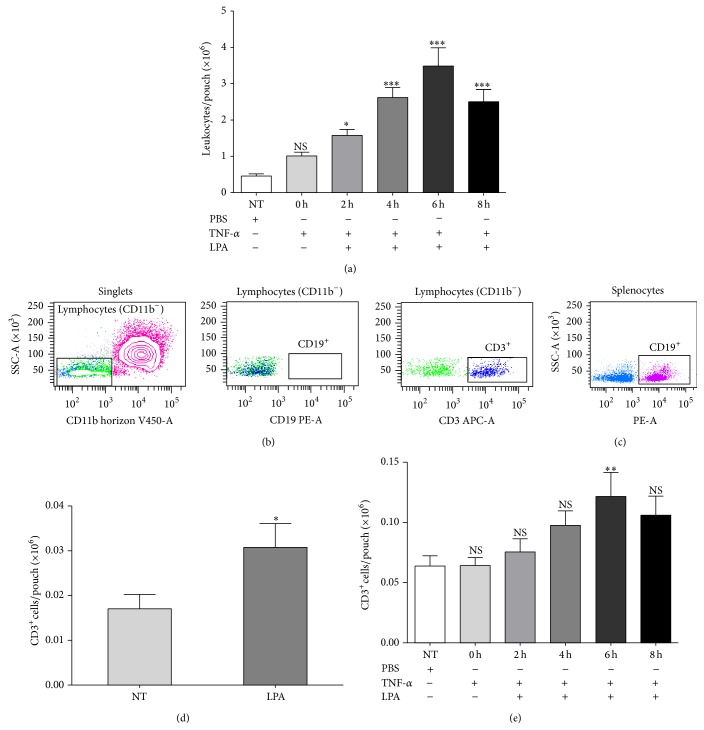
LPA-induced leukocyte recruitment in untreated and TNF-*α*-primed air pouches. (a) Kinetics of LPA-mediated leukocyte recruitment into TNF-*α*-treated air pouches. TNF-*α* (50 ng) was injected into the air pouches 16 h prior to stimulation with 3 *μ*g LPA for the indicated times. Air pouch exudates were collected and the number of leukocytes was determined as described in Section 2. (b) Leukocyte populations in lavage fluids collected from air pouches pretreated with TNF-*α* for 16 h and injected with LPA for 6 h. Cells were stained with various leukocyte markers and analyzed by flow cytometry. The CD11b^−^ cells were defined as lymphocytes according to their low granularity (left panel), which stained positive for CD3 (T cells, right panel) or CD19 (B cells, middle panel). (c) Labelling of B cells isolated from mouse spleen. Splenocytes were prepared as described in Section 2 and used for titration of anti-CD3e and anti-CD19 antibodies. (d) LPA-induced CD3^+^ cell recruitment into the air pouches. Air pouch exudates were collected at 6 h after LPA injection. The total number of leukocytes was measured and that of T cells determined by flow cytometry. (e) The absolute numbers of CD3^+^ cells recruited by LPA into TNF-*α*-primed air pouches was evaluated as described in (d). Data are the mean ± SE from 6 mice/group. ^*∗*^ *P* < 0.05, ^*∗∗*^ *P* < 0.01, and ^*∗∗∗*^ *P* < 0.001 by analysis of variance. SSC: side scatter; FSC: forward scatter.

Since CXCL13 is a ligand for CXCR5, a chemokine receptor expressed by mature B cells [19], and a subset of CD4^+^ and CD8^+^ T cells [20], we next determined whether LPA-mediated CXCL13 secretion contributes to recruitment of leukocyte subsets toward LPA into TNF-*α*-pretreated air pouches. As reported previously for LPA alone [11], LPA injected in TNF-*α*-pretreated air pouches (16 hours) stimulated the recruitment of leukocytes in a time-dependent manner (Figure 3(a)). An increase in the number of migrated leukocytes was detectable 2 h after LPA injection, peaked after 6 h, and declined thereafter. CD11b^+^ cells were the most prominent population in air pouch lavage fluids (Figure 3(b), left panel). We focused on CD11b^−^ cells and performed CD19-labelling to determine by FACS whether CD19^+^ B lymphocytes could be detected in air pouch exudates. Even though the CD19-PE antibody labeled B cells isolated from mouse spleens (Figure 3(c)), no CD19^+^ B lymphocytes were detected in air pouch exudates (Figure 3(b), middle panel). However, CD11b^−^/CD3^+^ cells were detected in air pouch lavage fluids (Figure 3(b), right panel).  Figure 3(d) shows that stimulation with LPA for 6 hours enhanced significantly the number of CD3^+^ cells in air pouch exudates (3.07 ± 0.53 × 10^4^ cells, 1.67 ± 0.03% of total leukocytes, *n* = 10) compared to mice injected with vehicle alone (1.7 ± 0.32 × 10^4^ cells, 1.88 ± 0.01% of total leukocytes, *n* = 10). The number of CD3^+^ cells in air pouch lavage fluids collected from TNF-*α*-pretreated air pouches was not different from that of mice injected with the vehicle alone. Furthermore, LPA injected into TNF-*α*-primed air pouches stimulated the recruitment of CD3^+^ cells in a time-dependent manner (Figure 3(e)). As observed for total leukocytes, recruitment of CD3^+^ cells peaked at the 6-hour time point following injection of LPA into air pouches (1.21 ± 0.19 × 10^5^ cells, 2.5 ± 0.9% of total leukocytes, *n* = 5).

### 3.3. Effect of Blocking CXCL13 on LPA-Mediated CD3^+^ Cell Recruitment

**Figure 4:**
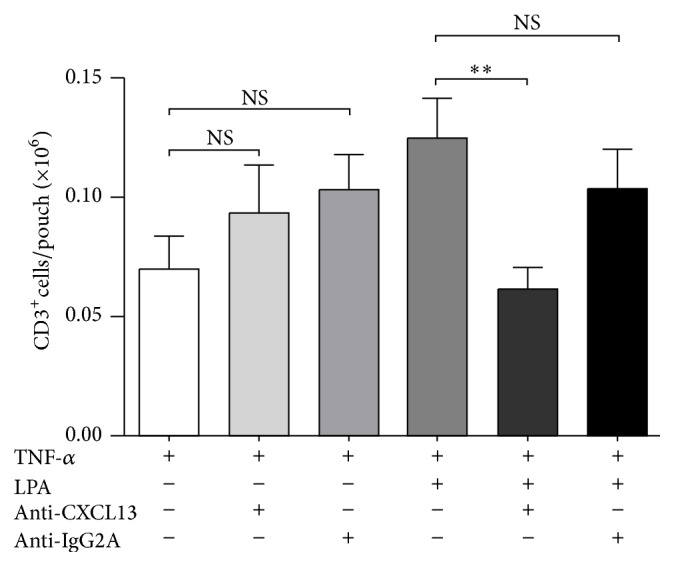
Effect of the CXCL13 neutralizing antibody on LPA-mediated lymphocyte recruitment into TNF-*α*-primed air pouches. Air pouch tissues were pretreated with TNF-*α* (50 ng) for 16 h prior to administration of LPA (3 *μ*g). Where indicated, the anti-CXCL13 neutralizing antibody or the isotype control antibody (IgG2A, 10 *μ*g) was administered into the air pouches 15 min prior to stimulation with LPA for 4 h. The absolute numbers of CD3^+^ T cells in air pouch lavage fluids were determined by flow cytometry as described in Section 2. Data are the mean ± SE of at least 5 mice/group. ^*∗∗*^ *P* < 0.01 by analysis of variance.

It was reported previously that antibody neutralization of CXCL13 can prevent migration of double-negative regulatory T lymphocytes to cardiac allografts implanted in the abdomen of mice [32]. To examine whether a correlation exists between CXCL13 secretion in response to LPA and CD3^+^ lymphocyte recruitment in our mouse model, a neutralizing anti-CXCL13 antibody was injected into TNF-*α*-primed air pouches prior to LPA stimulation. Injection of the neutralizing antibody against CXCL13 prior to LPA into the air pouch significantly reduced LPA-induced CD3^+^ lymphocyte recruitment into TNF-*α*-primed air pouches, whereas the isotype control antibody had no significant effect on LPA-mediated CD3^+^ lymphocyte influx (Figure 4). Taken together, the data indicate that CXCL13 plays a role in LPA-mediated recruitment of CD3^+^ lymphocytes into the air pouches.

## 4. Discussion

Extracellular LPA is a bioactive lysophospholipid produced by ATX that mediates its effects through activation of various LPA receptors [2]. Using the mouse air pouch model of inflammation, we previously demonstrated that LPA promotes the influx of neutrophils and other leukocyte subtypes including macrophages/monocytes and lymphocytes through activation of two LPA receptors, LPA1 and LPA3 [11]. Stimulation of these LPA receptors expressed by cells lining the air pouch cavity promotes the synthesis of various chemokines/cytokines (IL-6, IL-1*β*, IL-16, KC, IP-10, MIP-2, and CXCL13), the synthesis of which is greatly enhanced by TNF-*α* injected into the air pouches 16 hours prior to LPA. Although LPA-mediated KC synthesis was shown to play a predominant role in the recruitment of leukocytes into the air pouches, neutralization of KC or blocking of its cognate receptor CXCR2 was not able to totally abrogate the influx of leukocytes [11]. In the present study, we focused on CXCL13, a key chemoattractant of B cells and of subsets of T lymphocytes [19–21]. We report that administration of LPA or TNF-*α* into air pouches increased the levels of CXCL13 in air pouch lavage fluids in a time-dependent manner. The combination of a pretreatment of the air pouch tissues with TNF-*α* prior to LPA stimulation greatly enhanced LPA-mediated CXCL13 secretion. The release of CXCL13 induced by LPA peaked 2 hours ahead of the time point of maximal leukocyte recruitment, including that of CD3^+^ immune cells. Consistent with a role for CXCL13 in LPA-mediated CD3^+^ cell homing, antibody neutralization of CXCL13 prevented the influx of these T cells into TNF-*α*-pretreated air pouches.

Elevated levels of ATX and of LPA have been reported in synovial fluids collected from RA patients [4, 7–9]. The ATX-LPA axis is emerging as a regulator of lymphocyte homing and inflammation [33]. ATX binds to lymphocytes in a *α*4*β*1-dependent manner [34]. Through activation of LPA receptors expressed by T cells [33], LPA induces a polarized morphology that is required for transendothelium migration, a key step for regulation of naive T cell entry into secondary lymphoid organs [34, 35]. However, LPA does not promote directed cell migration* in vitro* but exerts a chemokinetic effect that increases the chemoattractant effect of chemokines regulating human and mouse T cell homing in various tissues [34–37]. We cannot exclude the possibility that LPA injected into air pouches has a motility-stimulating effect on leukocytes. However, we showed that CXCL13 secretion induced by LPA preceded the peak of leukocyte recruitment, including that of CD3^+^ cells by several hours. There was a twofold increase in the total number of CD3^+^cells in lavage fluids from TNF-*α*-pretreated air pouches after 6 hours of stimulation with LPA, but when expressed as a percentage of total leukocytes no increase in the relative abundance of CD3^+^ cells was noticed due to the massive recruitment of CD11b^+^ cells (i.e., neutrophils) at this time point [11]. Antibody neutralization of CXCL13 prior to LPA stimulation suggests that LPA was exerting its effect on CD3^+^ cell influx into TNF-*α*-pretreated air pouches in a manner that is dependent on CXCL13 synthesis. The lack of small molecule inhibitors of CXCR5 or CXCR5 neutralizing antibodies precluded further analyses of the molecular pathways by which LPA recruits CD3^+^ cells in this* in vivo* model of inflammation.

As CXCL13 is a ligand of CXCR5, which is expressed on B cells, macrophages, monocytes, double-negative Treg cells [32], and T helper cells in human and mouse [22, 38–40], it is possible that CXCL13 induced by LPA could play an important role in leukocyte homing in various diseases. CXCL13 was originally identified as a B cell chemoattractant [17]. Furthermore, lysophospholipids such as S1P and LPA have been suggested to regulate splenic B cell homing through CXCL13-mediated integrin-dependent adhesion [41]. Recruitment of B cells into the air pouch wall has been reported following stimulation with oxidized phospholipids and LPS [42]. In our experiments, after blocking Fc receptors with an anti-CD16/CD32 antibody, no B cells were detected in the lavage fluids collected after stimulation of TNF-*α*-pretreated air pouches with LPA. Whether B cells are recruited by LPA and remain sequestered in the air pouch wall will need further studies.

DCs [43], human monocytes/macrophages, and CD4^+^ T cell subsets are potent inducible sources of CXCL13 [44, 45]. We report that the basal levels of CXCL13 in air pouch lavage fluids increased quickly in response to LPA and well before an increase in immune cell influx could be monitored. CXCL13 secretion could be mediated through binding of LPA to its cognate receptors, possibly LPA1 and LPA3 [11], expressed by air pouch lining cells or discrete populations of cells recruited early. Although identification of cells that contribute to CXCL13 production awaits further characterization, this study suggests that a role for the CXCL13-CXCR5 axis in LPA-mediated regulation of immune cell trafficking to sites of inflammation cannot be ignored.

CXCL13 has been identified as a serologic marker predictive of disease severity in early RA [28, 29]. High levels of CXCL13 were measured in synovial fluids from RA patients, with RA synovial T helper cells contributing to CXCL13 secretion [46, 47]. Within the RA synovium CXCL13 is expressed in areas of B cell accumulation characteristic of ectopic lymphoid follicles where subtypes of CXCL13-expressing T cells (CD3^+^ and CD4^+^) and monocytes/macrophages colocalize [47–49]. The receptor for CXCL13 is upregulated in the RA synovium and associated with the presence of CXCR5 positive B cells and T cells infiltrating the synovia [22]. Of note Zheng et al. [50] reported that neutralization of CXCL13 at the boosting stage reduced the development of ectopic lymphoid follicles and the severity of collagen-induced arthritis in mice.

## 5. Conclusions

In summary, we provide evidence that LPA-induced CXCL13 secretion contributed to the recruitment of CD3^+^ T cells within the air pouch environment under conditions of inflammation exacerbated by TNF-*α*. This study extends the known role of CXCR2 ligand chemokines to the massive recruitment of leukocytes induced by LPA in this mouse model of inflammation [11]. Given that ATX-derived LPA plays a role in the pathogenesis of RA [7–9, 51], LPA-mediated CXCL13 secretion raises the question whether LPA contributes to the recruitment of lymphocytes and extranodal lymphoid neogenesis during chronic inflammation.

## Abbreviations

ATX: Autotaxin
DCs: Dendritic cells
LPA: Lysophosphatidic acid
CXCL13: C-X-C motif chemokine 13
CXCL8: C-X-C motif chemokine 8
CXCL1: C-X-C motif chemokine 1
GPCRs: G protein-coupled receptors
RA: Rheumatoid arthritis
TNF-*α*: Tumor necrosis factor-alpha.

## References

[1] Tokumura A. (1995). A family of phospholipid autacoids: occurrence, metabolism and bioactions. *Progress in Lipid Research*.

[2] Yung Y. C., Stoddard N. C., Chun J. (2014). LPA receptor signaling: pharmacology, physiology, and pathophysiology. *Journal of Lipid Research*.

[3] Kano K., Arima N., Ohgami M., Aoki J. (2008). LPA and its analogs-attractive tools for elucidation of LPA biology and drug development. *Current Medicinal Chemistry*.

[4] Bourgoin S. G., Zhao C. (2010). Autotaxin and lysophospholipids in rheumatoid arthritis. *Current Opinion in Investigational Drugs*.

[5] Zhao Y., Natarajan V. (2013). Lysophosphatidic acid (LPA) and its receptors: role in airway inflammation and remodeling. *Biochimica et Biophysica Acta*.

[6] Tigyi G. (2010). Aiming drug discovery at lysophosphatidic acid targets. *British Journal of Pharmacology*.

[7] Nochi H., Tomura H., Tobo M. (2008). Stimulatory role of lysophosphatidic acid in cyclooxygenase-2 induction by synovial fluid of patients with rheumatoid arthritis in fibroblast-like synovial cells. *Journal of Immunology*.

[8] Nikitopoulou I., Oikonomou N., Karouzakis E. (2012). Autotaxin expression from synovial fibroblasts is essential for the pathogenesis of modeled arthritis. *The Journal of Experimental Medicine*.

[9] Zhao C., Fernandes M. J., Prestwich G. D. (2008). Regulation of lysophosphatidic acid receptor expression and function in human synoviocytes: implications for rheumatoid arthritis?. *Molecular Pharmacology*.

[10] Houben A. J. S., Moolenaar W. H. (2011). Autotaxin and LPA receptor signaling in cancer. *Cancer and Metastasis Reviews*.

[11] Zhao C., Sardella A., Chun J., Poubelle P. E., Fernandes M. J., Bourgoin S. G. (2011). TNF-alpha promotes LPA1- and LPA3-mediated recruitment of leukocytes in vivo through CXCR2 ligand chemokines. *Journal of Lipid Research*.

[12] Zhao Y., Usatyuk P. V., Cummings R. (2005). Lipid phosphate phosphatase-1 regulates lysophosphatidic acid-induced calcium release, NF-*κ*B activation and interleukin-8 secretion in human bronchial epithelial cells. *Biochemical Journal*.

[13] Mu H., Calderone T. L., Davies M. A. (2012). Lysophosphatidic acid induces lymphangiogenesis and IL-8 production in vitro in human lymphatic endothelial cells. *American Journal of Pathology*.

[14] Aki Y., Kondo A., Nakamura H., Togari A. (2008). Lysophosphatidic acid-stimulated interleukin-6 and -8 synthesis through LPA1 receptors on human osteoblasts. *Archives of Oral Biology*.

[15] Schwartz B. M., Hong G., Morrison B. H. (2001). Lysophospholipids increase interleukin-8 expression in ovarian cancer cells. *Gynecologic Oncology*.

[16] Sallusto F., Baggiolini M. (2008). Chemokines and leukocyte traffic. *Nature Immunology*.

[17] Legler D. F., Loetscher M., Roos R. S., Clark-Lewis I., Baggiolini M., Moser B. (1998). B cell-attracting chemokine 1, a human CXC chemokine expressed in lymphoid tissues, selectively attracts B lymphocytes via BLR1/CXCR5. *Journal of Experimental Medicine*.

[18] Zlotnik A., Yoshie O., Nomiyama H. (2006). The chemokine and chemokine receptor superfamilies and their molecular evolution. *Genome Biology*.

[19] Muller G., Hopken U. E., Lipp M. (2003). The impact of CCR7 and CXCR5 on lymphoid organ development and systemic immunity. *Immunological Reviews*.

[20] Schaerli P., Willimann K., Lang A. B., Lipp M., Loetscher P., Moser B. (2000). CXC chemokine receptor 5 expression defines follicular homing T cells with B cell helper function. *Journal of Experimental Medicine*.

[21] Howard O. M. Z., Hui F. D., Shao B. S. (2005). Autoantigens signal through chemokine receptors: Uveitis antigens induce CXCR3- and CXCR5-expressing lymphocytes and immature dendritic cells to migrate. *Blood*.

[22] Schmutz C., Hulme A., Burman A. (2005). Chemokine receptors in the rheumatoid synovium: upregulation of CXCR5. *Arthritis Research & Therapy*.

[23] Arnold C. N., Campbell D. J., Lipp M., Butcher E. C. (2007). The germinal center response is impaired in the absence of T cell-expressed CXCR5. *European Journal of Immunology*.

[24] Romagnani P., Lasagni L., Annunziato F., Serio M., Romagnani S. (2004). CXC chemokines: the regulatory link between inflammation and angiogenesis. *Trends in Immunology*.

[25] Jenh C.-H., Cox M. A., Hipkin W. (2001). Human B cell-attracting chemokine 1 (BCA-1; CXCL13) is an agonist for the human CXCR3 receptor. *Cytokine*.

[26] Gelderblom H., Londoño D., Bai Y. (2007). High production of CXCL13 in blood and brain during persistent infection with the relapsing fever spirochete Borrelia turicatae. *Journal of Neuropathology and Experimental Neurology*.

[27] Rupprecht T. A., Pfister H.-W., Angele B., Kastenbauer S., Wilske B., Koedel U. (2005). The chemokine CXCL13 (BLC): a putative diagnostic marker for neuroborreliosis. *Neurology*.

[28] Meeuwisse C. M., van der Linden M. P., Rullmann T. A. (2011). Identification of CXCL13 as a marker for rheumatoid arthritis outcome using an in silico model of the rheumatic joint. *Arthritis & Rheumatism*.

[29] Bugatti S., Manzo A., Vitolo B. (2014). High expression levels of the B cell chemoattractant CXCL13 in rheumatoid synovium are a marker of severe disease. *Rheumatology*.

[30] Rupprecht T. A., Lechner C., Tumani H., Fingerle V. (2014). CXCL13: a biomarker for acute Lyme neuroborreliosis. Investigation of the predictive value in the clinical routine. *Nervenarzt*.

[31] Lisignoli G., Cristino S., Toneguzzi S. (2004). IL1*β* and TNF*α* differently modulate CXCL13 chemokine in stromal cells and osteoblasts isolated from osteoarthritis patients: evidence of changes associated to cell maturation. *Experimental Gerontology*.

[32] Lee B. P.-L., Chen W., Shi H., Der S. D., Förster R., Zhang L. (2006). CXCR5/CXCL13 interaction is important for double-negative regulatory T cell homing to cardiac allografts. *The Journal of Immunology*.

[33] Knowlden S., Georas S. N. (2014). The autotaxin-lpa axis emerges as a novel regulator of lymphocyte homing and inflammation. *Journal of Immunology*.

[34] Kanda H., Newton R., Klein R., Morita Y., Gunn M. D., Rosen S. D. (2008). Autotaxin, an ectoenzyme that produces lysophosphatidic acid, promotes the entry of lymphocytes into secondary lymphoid organs. *Nature Immunology*.

[35] Zhang Y., Chen Y.-C. M., Krummel M. F., Rosen S. D. (2012). Autotaxin through lysophosphatidic acid stimulates polarization, motility, and transendothelial migration of naive T cells. *Journal of Immunology*.

[36] Zheng Y., Kong Y., Goetzl E. J. (2001). Lysophosphatidic acid receptor-selective effects on Jurkat T cell migration through a matrigel model basement membrane. *The Journal of Immunology*.

[37] Kotarsky K., Boketoft Å., Bristulf J. (2006). Lysophosphatidic acid binds to and activates GPR92, a G protein-coupled receptor highly expressed in gastrointestinal lymphocytes. *The Journal of Pharmacology and Experimental Therapeutics*.

[38] Iyer S. S., Latner D. R., Zilliox M. J. (2013). Identification of novel markers for mouse CD4^+^ T follicular helper cells. *European Journal of Immunology*.

[39] Slight S. R., Rangel-Moreno J., Gopal R. (2013). CXCR5^+^ T helper cells mediate protective immunity against tuberculosis. *The Journal of Clinical Investigation*.

[40] Chevalier N., Jarrossay D., Ho E. (2011). CXCR5 expressing human central memory CD4 T cells and their relevance for humoral immune responses. *The Journal of Immunology*.

[41] Rieken S., Herroeder S., Sassmann A. (2006). Lysophospholipids control integrin-dependent adhesion in splenic B cells through G_i_ and G_12_/G_13_ family G-proteins but not through G_q_/G_11_. *Journal of Biological Chemistry*.

[42] Kadl A., Galkina E., Leitinger N. (2009). Induction of CCR2-dependent macrophage accumulation by oxidized phospholipids in the air-pouch model of inflammation. *Arthritis & Rheumatism*.

[43] Vissers J. L., Hartgers F. C., Lindhout E., Figdor C. G., Adema G. J. (2001). BLC (CXCL13) is expressed by different dendritic cell subsets in vitro and in vivo. *European Journal of Immunology*.

[44] Carlsen H. S., Baekkevold E. S., Morton H. C., Haraldsen G., Brandtzaeg P. (2004). Monocyte-like and mature macrophages produce CXCL13 (B cell-attracting chemokine 1) in inflammatory lesions with lymphoid neogenesis. *Blood*.

[45] Kobayashi S., Murata K., Shibuya H. (2013). A distinct human CD4+ T cell subset that secretes CXCL13 in rheumatoid synovium. *Arthritis and Rheumatism*.

[46] Takagi R., Higashi T., Hashimoto K. (2008). B Cell chemoattractant CXCL13 is preferentially expressed by human Th17 cell clones. *The Journal of Immunology*.

[47] Manzo A., Vitolo B., Humby F. (2008). Mature antigen-experienced T helper cells synthesize and secrete the B cell chemoattractant CXCL13 in the inflammatory environment of the rheumatoid joint. *Arthritis and Rheumatism*.

[48] Shi K., Hayashida K., Kaneko M. (2001). Lymphoid chemokine B cell-attracting chemokine-1 (CXCL13) is expressed in germinal center of ectopic lymphoid follicles within the synovium of chronic arthritis patients. *The Journal of Immunology*.

[49] Takemura S., Braun A., Crowson C. (2001). Lymphoid neogenesis in rheumatoid synovitis. *The Journal of Immunology*.

[50] Zheng B., Ozen Z., Zhang X. (2005). CXCL13 neutralization reduces the severity of collagen-induced arthritis. *Arthritis and Rheumatism*.

[51] Orosa B., García S., Martínez P., González A., Gómez-Reino J. J., Conde C. (2014). Lysophosphatidic acid receptor inhibition as a new multipronged treatment for rheumatoid arthritis. *Annals of the Rheumatic Diseases*.

